# Poly[[μ-1,4-bis­(imidazol-1-ylmeth­yl)benzene]bis­(μ_4_-cyclo­hexane-1,4-dicarboxyl­ato)dinickel(II)]

**DOI:** 10.1107/S1600536809029249

**Published:** 2009-07-31

**Authors:** Bing-Bing Li, Gai-Xia Fang, Xiao-Na Ji, Bo Xiao, Edward R. T. Tiekink

**Affiliations:** a Department of Bioengineering, Henan University of Urban Construction, Pingdingshan 467000, People’s Republic of China; b School of Environmental Science and Engineering, Huazhong University of Science and Technology, Wuhan 430074, People’s Republic of China; c Department of Chemistry, Universidade Federal de São Carlos, 13565-905 São Carlos, SP, Brazil

## Abstract

The structure of the polymeric title compound, [Ni_2_(C_8_H_10_O_4_)_2_(C_14_H_14_N_4_)]_*n*_, features a five-coordinate Ni^II^ centre defined by four carboxyl­ate O atoms from two different cyclo­hexane-1,4-dicarboxyl­ate (chdc) ligands and an N atom from one end of a 1,4-bis­(imidazol-1-ylmeth­yl)benzene (1,4-bix) mol­ecule. The NO_4_ coordination geometry is distorted square-pyramidal with the N atom in the apical position. Each end of the chdc ligand links pairs of Ni^II^ atoms into a paddle-wheel assembly, *i.e.* Ni_2_(O_2_C*R*′)_4_. These are connected into rows owing to the bridging nature of the chdc ligands, and the rows are connected into a two-dimensional grid *via* the 1,4-bix ligands. The 1,4-bix ligand, which is disposed about a centre of inversion, is disorderd. Two positions of equal occupancy were discerned for the –H_2_C(C_6_H_4_)CH_2_– residue.

## Related literature

For background to coordination polymers, see: Batten & Robson (1998 ▶); Kim & Jung (2002 ▶); Yang *et al.* (2008 ▶). For a related Ni(II) structure, see: Lee *et al.* (2003 ▶).
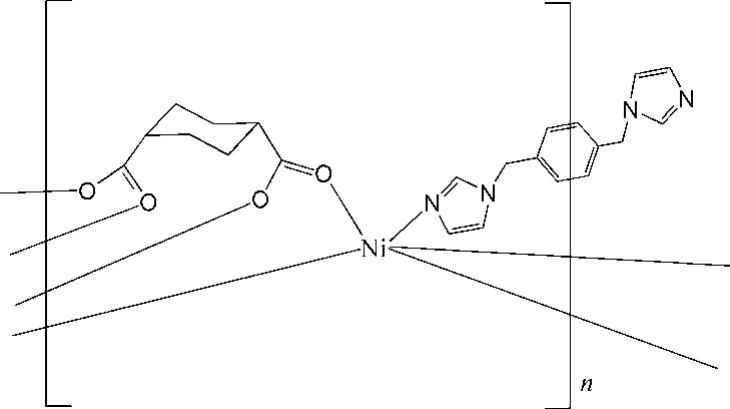


## Experimental

### 

#### Crystal data

[Ni_2_(C_8_H_10_O_4_)_2_(C_14_H_14_N_4_)]
*M* *_r_* = 696.03
Triclinic, 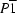

*a* = 8.4966 (6) Å
*b* = 8.8076 (6) Å
*c* = 10.7327 (8) Å
α = 93.567 (6)°
β = 100.608 (6)°
γ = 105.807 (6)°
*V* = 754.22 (9) Å^3^
*Z* = 1
Mo *K*α radiation
μ = 1.31 mm^−1^
*T* = 293 K
0.31 × 0.22 × 0.18 mm

#### Data collection

Bruker SMART APEX diffractometer
Absorption correction: multi-scan (*SADABS*; Sheldrick, 1996 ▶) *T*_min_ = 0.557, *T*_max_ = 0.791
6115 measured reflections
2640 independent reflections
2287 reflections with *I* > 2σ(*I*)
*R*_int_ = 0.025

#### Refinement

*R*[*F* ^2^ > 2σ(*F* ^2^)] = 0.052
*wR*(*F* ^2^) = 0.139
*S* = 1.11
2640 reflections
224 parameters
36 restraints
H-atom parameters constrained
Δρ_max_ = 1.30 e Å^−3^
Δρ_min_ = −1.25 e Å^−3^

### 

Data collection: *SMART* (Bruker, 1997 ▶); cell refinement: *SAINT* (Bruker, 1999 ▶); data reduction: *SAINT*; program(s) used to solve structure: *SHELXS97* (Sheldrick, 2008 ▶); program(s) used to refine structure: *SHELXL97* (Sheldrick, 2008 ▶); molecular graphics: *DIAMOND* (Brandenburg, 2006 ▶); software used to prepare material for publication: *SHELXL97*.

## Supplementary Material

Crystal structure: contains datablocks global, I. DOI: 10.1107/S1600536809029249/ng2618sup1.cif

Structure factors: contains datablocks I. DOI: 10.1107/S1600536809029249/ng2618Isup2.hkl

Additional supplementary materials:  crystallographic information; 3D view; checkCIF report

## Figures and Tables

**Fig. 1.**
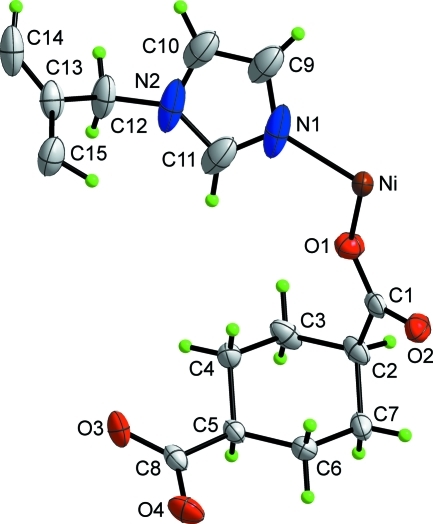
The asymmetric unit in the polymeric structure of (I), showing the atomic numbering scheme. Displacement ellipsoids are drawn at the 50% probability level. Only one component of the disordered -CH2(C6H4)CH2- residue is shown.

**Fig. 2.**
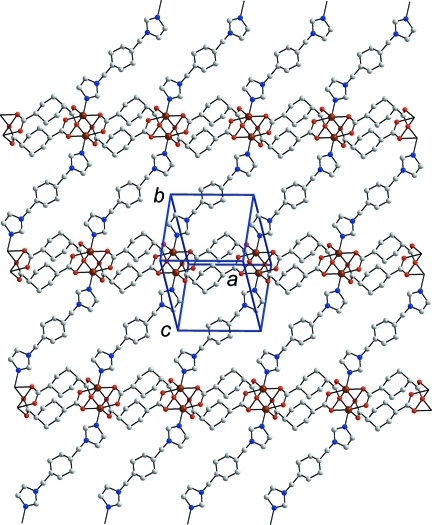
View of the 2-D array in (I). H atoms have been omitted for clarity.

**Fig. 3.**
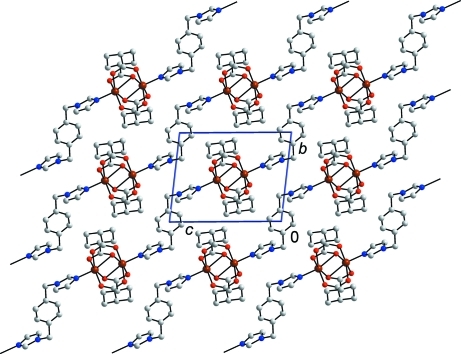
View of the stacking of the layers in the crystal structure of (I). H atoms have been omitted for clarity.

## supplementary crystallographic information

### Comment

Metal–organic coordination polymers continue to attract considerable interest owing to their well documented and varied applications (Yang *et al.*,  2008). These coordination polymers can be specially designed by the careful selection of metal cations with preferred coordination geometries, the nature of the anions, the structure of the connecting ligands, and the reaction conditions (Kim & Jung, 2002). The selection of ligand is extremely important because changing their geometries can control the topologies of the resulting coordination frameworks. While the rigid rod-like spacer, 4,4'-bipyridine, is well known in the construction of metal-organic polymers, flexible N-donor ligands such as 1,4-bis(imidazole-1-ylmethyl)benzene (1,4-bix) have not been so well explored. In this work, 1,4-bix assembles with nickel cyclohexane-1,4-dicarboxylate (chdc) to furnish [Ni(chdc)(1,4-bix)_0.5_], (I), which exists as a 2-D array.

The asymmetric unit of (I) comprises a Ni atom, a chdc dianion, and half a 1-4-bix molecule which is disposed about a centre of inversion (Fig. 1). Each end of the chdc ligand bridges a pair of Ni atoms to result in the formation of a paddle-wheel assembly, i.e. Ni_2_(O_2_CR')_4_. These are linked into rows which, in turn, are linked via the bridging 1,4-bix ligands into a 2-D array in the bc plane (Fig. 2). The layers are stacked in an ···ABC··· fashion (Fig. 3). The coordination geometry is based on a NO_4_ donor set that defines a square pyramid with the N donor atom in the apical position. If the second Ni atom in the paddle-wheel assembly is considered as occupying a coordination site, the Ni···Ni^i^ distance is 2.6529 (10) Å, the coordination geometry would be distorted octahedral; symmetry operation i: 2-x, 1-y, 1-z.

### Experimental

Nickel chloride hexahydrate (0.118 g, 0.5 mmol), H_2_chdc (0.135 g, 0.5 mmol) and 1,4-bix (0.093 g, 0.5 mmol) were placed in water (12 ml), and triethylamine was added until the pH value of the solution was 5.7. The solution was heated in a 23-ml Teflon-lined stainless-steel autoclave at 440 K for 5 days. The autoclave was allowed to cool to room temperature over several hours. Green blocks were isolated in about 61% yield.

### Refinement

Carbon-bound H-atoms were placed in calculated positions with C—H = 0.93 - 0.98 Å, and were included in the refinement in the riding model approximation, with *U*(H) set to 1.2*U*_eq_(C).

Disorder was noted in bridging 1,4-bix molecule. Two positions of equal weight (from refinement) were discerned for the -H_2_C(C_6_H_4)_CH_2_- residue but not for the imidazole ring, although several of the atoms exhibited elongated displacement ellipsoids. The atoms of this ring were restrained to be approximately isotropic with application of the ISOR command in SHELXL-97 (Sheldrick, 2008).

The maximum and minimum residual electron density peaks of 1.30 and -1.25 eÅ^-3^, respectively, were located 0.95 Å and 1.58 Å from the C26 and H13 atoms, respectively.

### Crystal data

**Table:**

[Ni_2_(C_8_H_10_O_4_)_2_(C_14_H_14_N_4_)]	*Z* = 1
*M**_r_* = 696.03	*F*(000) = 362
Triclinic, *P*1	*D*_x_ = 1.532 Mg m^−^^3^
Hall symbol: -P 1	Mo *K*α radiation, λ = 0.71073 Å
*a* = 8.4966 (6) Å	Cell parameters from 3051 reflections
*b* = 8.8076 (6) Å	θ = 3.0–26.4°
*c* = 10.7327 (8) Å	µ = 1.31 mm^−^^1^
α = 93.567 (6)°	*T* = 293 K
β = 100.608 (6)°	Block, green
γ = 105.807 (6)°	0.31 × 0.22 × 0.18 mm
*V* = 754.22 (9) Å^3^	

### Data collection

**Table:**

Bruker SMART APEX diffractometer	2640 independent reflections
Radiation source: fine-focus sealed tube	2287 reflections with *I* > 2σ(*I*)
graphite	*R*_int_ = 0.025
φ and ω scans	θ_max_ = 25.0°, θ_min_ = 4.3°
Absorption correction: multi-scan (*SADABS*; Sheldrick, 1996)	*h* = −10→10
*T*_min_ = 0.557, *T*_max_ = 0.791	*k* = −10→10
6115 measured reflections	*l* = −12→12

### Refinement

**Table:**

Refinement on *F*^2^	Primary atom site location: structure-invariant direct methods
Least-squares matrix: full	Secondary atom site location: difference Fourier map
*R*[*F*^2^ > 2σ(*F*^2^)] = 0.052	Hydrogen site location: inferred from neighbouring sites
*wR*(*F*^2^) = 0.139	H-atom parameters constrained
*S* = 1.11	*w* = 1/[σ^2^(*F*_o_^2^) + (0.0653*P*)^2^ + 2.0659*P*] where *P* = (*F*_o_^2^ + 2*F*_c_^2^)/3
2640 reflections	(Δ/σ)_max_ < 0.001
224 parameters	Δρ_max_ = 1.30 e Å^−^^3^
36 restraints	Δρ_min_ = −1.25 e Å^−^^3^

### Special details

**Table:**

Geometry. All s.u.'s (except the s.u. in the dihedral angle between two l.s. planes) are estimated using the full covariance matrix. The cell s.u.'s are taken into account individually in the estimation of s.u.'s in distances, angles and torsion angles; correlations between s.u.'s in cell parameters are only used when they are defined by crystal symmetry. An approximate (isotropic) treatment of cell s.u.'s is used for estimating s.u.'s involving l.s. planes.
Refinement. Refinement of F^2^ against ALL reflections. The weighted R-factor wR and goodness of fit S are based on F^2^, conventional R-factors R are based on F, with F set to zero for negative F^2^. The threshold expression of F^2^ > 2σ(F^2^) is used only for calculating R-factors(gt) etc. and is not relevant to the choice of reflections for refinement. R-factors based on F^2^ are statistically about twice as large as those based on F, and R- factors based on ALL data will be even larger.

### Fractional atomic coordinates and isotropic or equivalent isotropic displacement parameters (Å^2^)

**Table:**

	*x*	*y*	*z*	*U*_iso_*/*U*_eq_	Occ. (<1)
Ni	1.02456 (6)	0.46763 (6)	0.61988 (5)	0.0239 (2)	
O1	0.9631 (4)	0.6690 (4)	0.6585 (3)	0.0338 (7)	
O2	0.9113 (4)	0.7154 (4)	0.4545 (3)	0.0335 (7)	
O3	0.2691 (4)	0.5884 (4)	0.6790 (3)	0.0397 (8)	
O4	0.2180 (4)	0.6331 (4)	0.4772 (3)	0.0418 (8)	
N1	0.9779 (5)	0.3729 (5)	0.7773 (4)	0.0437 (8)	
C1	0.9120 (5)	0.7449 (5)	0.5710 (4)	0.0264 (9)	
C2	0.8505 (5)	0.8851 (5)	0.6088 (4)	0.0297 (10)	
H2	0.9473	0.9801	0.6264	0.036*	
C3	0.7810 (5)	0.8673 (6)	0.7302 (4)	0.0358 (11)	
H3A	0.8616	0.8425	0.7962	0.043*	
H3B	0.7655	0.9675	0.7597	0.043*	
C4	0.6153 (5)	0.7376 (6)	0.7096 (4)	0.0323 (10)	
H4A	0.5740	0.7329	0.7882	0.039*	
H4B	0.6319	0.6356	0.6870	0.039*	
C5	0.4869 (5)	0.7705 (5)	0.6037 (4)	0.0245 (9)	
H5	0.4763	0.8750	0.6305	0.029*	
C6	0.5532 (5)	0.7863 (6)	0.4803 (4)	0.0298 (10)	
H6A	0.5667	0.6854	0.4501	0.036*	
H6B	0.4726	0.8126	0.4152	0.036*	
C7	0.7204 (5)	0.9147 (6)	0.5016 (5)	0.0340 (11)	
H7A	0.7040	1.0172	0.5226	0.041*	
H7B	0.7620	0.9179	0.4231	0.041*	
C8	0.3134 (5)	0.6545 (5)	0.5852 (4)	0.0287 (10)	
C9	1.0595 (8)	0.2825 (7)	0.8462 (5)	0.0573 (9)	
H9	1.1499	0.2520	0.8274	0.069*	
C10	0.9880 (8)	0.2448 (7)	0.9457 (6)	0.0573 (9)	
H10	1.0191	0.1824	1.0070	0.069*	
C11	0.8601 (8)	0.3888 (7)	0.8366 (5)	0.0573 (9)	
H11	0.7842	0.4449	0.8098	0.069*	
N2	0.8646 (6)	0.3123 (5)	0.9419 (4)	0.0437 (8)	0.50
C12	0.782 (2)	0.287 (2)	1.0446 (17)	0.047 (4)	0.50
H12A	0.7309	0.3719	1.0558	0.057*	0.50
H12B	0.8638	0.2931	1.1222	0.057*	0.50
C13	0.645 (2)	0.125 (2)	1.025 (2)	0.037 (4)	0.50
C14	0.653 (4)	0.031 (4)	1.109 (3)	0.063 (7)	0.50
H14	0.7448	0.0329	1.1717	0.075*	0.50
C15	0.520 (3)	0.084 (3)	0.916 (2)	0.050 (5)	0.50
H15	0.5460	0.1367	0.8469	0.060*	0.50
N2'	0.8646 (6)	0.3123 (5)	0.9419 (4)	0.0437 (8)	0.50
C12'	0.716 (2)	0.324 (2)	1.0150 (16)	0.059 (5)	0.50
H12C	0.7636	0.3649	1.1041	0.071*	0.50
H12D	0.6598	0.3975	0.9768	0.071*	0.50
C13'	0.594 (3)	0.164 (2)	1.005 (2)	0.045 (5)	0.50
C14'	0.606 (3)	0.063 (4)	1.104 (3)	0.057 (8)	0.50
H14'	0.6670	0.1156	1.1832	0.068*	0.50
C15'	0.464 (3)	0.114 (3)	0.898 (3)	0.055 (5)	0.50
H15'	0.4271	0.1766	0.8397	0.066*	0.50

### Atomic displacement parameters (Å^2^)

**Table:**

	*U*^11^	*U*^22^	*U*^33^	*U*^12^	*U*^13^	*U*^23^
Ni	0.0223 (3)	0.0278 (3)	0.0238 (3)	0.0071 (2)	0.0085 (2)	0.0080 (2)
O1	0.0353 (17)	0.0345 (18)	0.0354 (17)	0.0157 (14)	0.0079 (14)	0.0050 (14)
O2	0.0375 (18)	0.0329 (18)	0.0368 (18)	0.0149 (14)	0.0161 (14)	0.0087 (14)
O3	0.0254 (16)	0.043 (2)	0.049 (2)	0.0002 (14)	0.0171 (15)	0.0070 (16)
O4	0.0239 (16)	0.044 (2)	0.049 (2)	0.0069 (15)	−0.0055 (15)	−0.0014 (16)
N1	0.0501 (18)	0.0403 (17)	0.0287 (15)	−0.0138 (14)	0.0191 (13)	0.0039 (13)
C1	0.0152 (18)	0.024 (2)	0.039 (3)	0.0019 (16)	0.0096 (17)	0.0062 (19)
C2	0.0181 (19)	0.022 (2)	0.047 (3)	0.0016 (17)	0.0077 (18)	0.0006 (19)
C3	0.024 (2)	0.046 (3)	0.033 (2)	0.010 (2)	−0.0002 (18)	−0.010 (2)
C4	0.024 (2)	0.049 (3)	0.026 (2)	0.012 (2)	0.0081 (18)	0.010 (2)
C5	0.0177 (19)	0.026 (2)	0.031 (2)	0.0065 (17)	0.0069 (17)	0.0043 (17)
C6	0.024 (2)	0.038 (3)	0.030 (2)	0.0124 (19)	0.0068 (17)	0.0096 (19)
C7	0.028 (2)	0.034 (3)	0.050 (3)	0.014 (2)	0.021 (2)	0.017 (2)
C8	0.020 (2)	0.028 (2)	0.041 (3)	0.0090 (18)	0.0101 (19)	0.0035 (19)
C9	0.065 (2)	0.050 (2)	0.0432 (18)	−0.0045 (16)	0.0031 (16)	0.0189 (15)
C10	0.065 (2)	0.050 (2)	0.0432 (18)	−0.0045 (16)	0.0031 (16)	0.0189 (15)
C11	0.065 (2)	0.050 (2)	0.0432 (18)	−0.0045 (16)	0.0031 (16)	0.0189 (15)
N2	0.0501 (18)	0.0403 (17)	0.0287 (15)	−0.0138 (14)	0.0191 (13)	0.0039 (13)
C12	0.048 (10)	0.042 (8)	0.041 (8)	−0.010 (6)	0.020 (7)	0.003 (6)
C13	0.041 (9)	0.033 (10)	0.036 (8)	0.002 (6)	0.021 (7)	−0.006 (6)
C14	0.058 (15)	0.064 (14)	0.065 (11)	−0.004 (10)	0.041 (10)	0.009 (10)
C15	0.057 (14)	0.056 (11)	0.039 (9)	0.008 (9)	0.022 (10)	0.013 (8)
N2'	0.0501 (18)	0.0403 (17)	0.0287 (15)	−0.0138 (14)	0.0191 (13)	0.0039 (13)
C12'	0.070 (13)	0.054 (11)	0.040 (10)	−0.020 (8)	0.044 (9)	−0.016 (7)
C13'	0.064 (13)	0.031 (9)	0.039 (9)	−0.007 (7)	0.042 (10)	−0.001 (7)
C14'	0.050 (13)	0.071 (15)	0.035 (9)	−0.013 (10)	0.030 (9)	−0.026 (9)
C15'	0.047 (12)	0.058 (13)	0.056 (12)	0.001 (8)	0.022 (10)	0.010 (8)

### Geometric parameters (Å, °)

**Table:**

Ni—N1	1.987 (4)	C7—H7A	0.9700
Ni—O2^i^	2.003 (3)	C7—H7B	0.9700
Ni—O3^ii^	2.019 (3)	C9—C10	1.339 (8)
Ni—O1	2.021 (3)	C9—H9	0.9300
Ni—O4^iii^	2.054 (3)	C10—N2	1.334 (8)
Ni—Ni^i^	2.6529 (10)	C10—N2'	1.334 (8)
O1—C1	1.266 (5)	C10—H10	0.9300
O2—C1	1.260 (5)	C11—N2	1.352 (7)
O2—Ni^i^	2.003 (3)	C11—N2'	1.352 (7)
O3—C8	1.260 (6)	C11—H11	0.9300
O3—Ni^iv^	2.019 (3)	N2—C12	1.409 (18)
O4—C8	1.256 (6)	C12—C13	1.55 (3)
O4—Ni^iii^	2.054 (3)	C12—H12A	0.9700
N1—C11	1.314 (8)	C12—H12B	0.9700
N1—C9	1.360 (8)	C13—C14	1.27 (5)
C1—C2	1.526 (6)	C13—C15	1.38 (3)
C2—C3	1.526 (6)	C14—C15^v^	1.50 (4)
C2—C7	1.530 (6)	C14—H14	0.9300
C2—H2	0.9800	C15—C14^v^	1.50 (4)
C3—C4	1.521 (6)	C15—H15	0.9300
C3—H3A	0.9700	N2'—C12'	1.626 (16)
C3—H3B	0.9700	C12'—C13'	1.49 (3)
C4—C5	1.524 (6)	C12'—H12C	0.9700
C4—H4A	0.9700	C12'—H12D	0.9700
C4—H4B	0.9700	C13'—C15'	1.39 (4)
C5—C8	1.517 (6)	C13'—C14'	1.43 (5)
C5—C6	1.531 (6)	C14'—C15'^v^	1.50 (5)
C5—H5	0.9800	C14'—H14'	0.9300
C6—C7	1.525 (6)	C15'—C14'^v^	1.50 (5)
C6—H6A	0.9700	C15'—H15'	0.9300
C6—H6B	0.9700		
			
N1—Ni—O2^i^	95.33 (16)	C6—C7—H7B	109.2
N1—Ni—O3^ii^	100.50 (16)	C2—C7—H7B	109.2
O2^i^—Ni—O3^ii^	89.68 (14)	H7A—C7—H7B	107.9
N1—Ni—O1	96.72 (16)	O4—C8—O3	122.9 (4)
O2^i^—Ni—O1	167.83 (12)	O4—C8—C5	118.1 (4)
O3^ii^—Ni—O1	89.76 (14)	O3—C8—C5	119.0 (4)
N1—Ni—O4^iii^	92.29 (16)	C10—C9—N1	108.5 (6)
O2^i^—Ni—O4^iii^	89.74 (14)	C10—C9—H9	125.7
O3^ii^—Ni—O4^iii^	167.19 (14)	N1—C9—H9	125.7
O1—Ni—O4^iii^	88.12 (13)	N2—C10—N2	0.00 (18)
N1—Ni—Ni^i^	159.62 (13)	N2—C10—C9	108.3 (5)
O2^i^—Ni—Ni^i^	83.45 (9)	N2'—C10—C9	108.3 (5)
O3^ii^—Ni—Ni^i^	99.83 (10)	N2—C10—H10	125.9
O1—Ni—Ni^i^	84.67 (9)	N2'—C10—H10	125.9
O4^iii^—Ni—Ni^i^	67.40 (10)	C9—C10—H10	125.9
C1—O1—Ni	122.0 (3)	N1—C11—N2	110.5 (6)
C1—O2—Ni^i^	124.7 (3)	N1—C11—N2'	110.5 (6)
C8—O3—Ni^iv^	106.2 (3)	N2—C11—N2	0.0 (3)
C8—O4—Ni^iii^	143.4 (3)	N1—C11—H11	124.7
C11—N1—C9	106.2 (5)	N2—C11—H11	124.7
C11—N1—Ni	125.6 (4)	N2'—C11—H11	124.7
C9—N1—Ni	128.2 (4)	C10—N2—C11	106.4 (5)
O2—C1—O1	124.8 (4)	C10—N2—C12	114.4 (8)
O2—C1—C2	117.1 (4)	C11—N2—C12	139.1 (8)
O1—C1—C2	118.1 (4)	N2—C12—C13	112.9 (15)
C1—C2—C3	112.5 (4)	N2—C12—H12A	109.0
C1—C2—C7	112.5 (4)	C13—C12—H12A	109.0
C3—C2—C7	109.7 (3)	N2'—C12—H12B	109.0
C1—C2—H2	107.3	C13—C12—H12B	109.0
C3—C2—H2	107.3	H12A—C12—H12B	107.8
C7—C2—H2	107.3	C14—C13—C15	121 (2)
C4—C3—C2	112.4 (4)	C14—C13—C12	119 (2)
C4—C3—H3A	109.1	C15—C13—C12	120.2 (18)
C2—C3—H3A	109.1	C13—C14—C15^v^	105 (3)
C4—C3—H3B	109.1	C13—C14—H14	127.7
C2—C3—H3B	109.1	C15^v^—C14—H14	127.7
H3A—C3—H3B	107.9	C13—C15—C14^v^	131 (3)
C3—C4—C5	110.5 (4)	C13—C15—H15	114.6
C3—C4—H4A	109.6	C14^v^—C15—H15	114.6
C5—C4—H4A	109.6	C10—N2—C11	106.4 (5)
C3—C4—H4B	109.6	C10—N2—C12'	141.6 (8)
C5—C4—H4B	109.6	C11—N2—C12'	111.8 (9)
H4A—C4—H4B	108.1	C13'—C12'—N2	109.7 (13)
C8—C5—C4	113.8 (4)	C13'—C12'—H12C	109.7
C8—C5—C6	113.3 (4)	N2'—C12'—H12C	109.7
C4—C5—C6	110.4 (3)	C13'—C12'—H12D	109.7
C8—C5—H5	106.2	N2'—C12'—H12D	109.7
C4—C5—H5	106.2	H12C—C12'—H12D	108.2
C6—C5—H5	106.2	C15'—C13'—C14'	119 (2)
C7—C6—C5	111.1 (4)	C15'—C13'—C12'	118.9 (18)
C7—C6—H6A	109.4	C14'—C13'—C12'	122 (2)
C5—C6—H6A	109.4	C13'—C14'—C15'^v^	131 (2)
C7—C6—H6B	109.4	C13'—C14'—H14'	114.4
C5—C6—H6B	109.4	C15'^v^—C14'—H14'	114.4
H6A—C6—H6B	108.0	C13'—C15'—C14'^v^	106 (2)
C6—C7—C2	112.0 (4)	C13'—C15'—H15'	126.8
C6—C7—H7A	109.2	C14'^v^—C15'—H15'	126.8
C2—C7—H7A	109.2		
			
N1—Ni—O1—C1	−153.4 (3)	C11—N1—C9—C10	0.4 (6)
O2^i^—Ni—O1—C1	18.7 (8)	Ni—N1—C9—C10	179.5 (4)
O3^ii^—Ni—O1—C1	106.0 (3)	N1—C9—C10—N2	−1.1 (7)
O4^iii^—Ni—O1—C1	−61.3 (3)	N1—C9—C10—N2'	−1.1 (7)
Ni^i^—Ni—O1—C1	6.1 (3)	C9—N1—C11—N2	0.4 (6)
O2^i^—Ni—N1—C11	−144.6 (5)	Ni—N1—C11—N2	−178.7 (3)
O3^ii^—Ni—N1—C11	124.7 (5)	C9—N1—C11—N2'	0.4 (6)
O1—Ni—N1—C11	33.7 (5)	Ni—N1—C11—N2'	−178.7 (3)
O4^iii^—Ni—N1—C11	−54.7 (5)	N2—C10—N2—C11	0(100)
Ni^i^—Ni—N1—C11	−59.1 (7)	C9—C10—N2—C11	1.3 (7)
O2^i^—Ni—N1—C9	36.5 (5)	N2—C10—N2'—C12	0(100)
O3^ii^—Ni—N1—C9	−54.2 (5)	C9—C10—N2'—C12	−177.6 (9)
O1—Ni—N1—C9	−145.2 (5)	N1—C11—N2—C10	−1.1 (6)
O4^iii^—Ni—N1—C9	126.4 (5)	N2—C11—N2—C10	0(100)
Ni^i^—Ni—N1—C9	122.0 (5)	N1—C11—N2'—C12	177.4 (12)
Ni^i^—O2—C1—O1	4.6 (6)	N2—C11—N2'—C12	0(100)
Ni^i^—O2—C1—C2	−177.0 (3)	C10—N2—C12—C13	−82.7 (13)
Ni—O1—C1—O2	−8.1 (6)	C11—N2—C12—C13	98.9 (15)
Ni—O1—C1—C2	173.4 (3)	N2—C12—C13—C14	124 (2)
O2—C1—C2—C3	153.7 (4)	N2—C12—C13—C15	−55 (2)
O1—C1—C2—C3	−27.8 (5)	C15—C13—C14—C15^v^	−19 (3)
O2—C1—C2—C7	29.2 (5)	C12—C13—C14—C15^v^	162.1 (17)
O1—C1—C2—C7	−152.3 (4)	C14—C13—C15—C14^v^	24 (4)
C1—C2—C3—C4	−70.5 (5)	C12—C13—C15—C14^v^	−157 (2)
C7—C2—C3—C4	55.6 (5)	N2—C10—N2—C11	0(100)
C2—C3—C4—C5	−57.3 (5)	C9—C10—N2—C11	1.3 (7)
C3—C4—C5—C8	−174.8 (4)	N2—C10—N2'—C12'	0(100)
C3—C4—C5—C6	56.5 (5)	C9—C10—N2'—C12'	176.8 (11)
C8—C5—C6—C7	174.9 (3)	N1—C11—N2—C10	−1.1 (6)
C4—C5—C6—C7	−56.2 (5)	N2—C11—N2—C10	0(100)
C5—C6—C7—C2	55.8 (5)	N1—C11—N2'—C12'	−178.1 (8)
C1—C2—C7—C6	71.4 (5)	N2—C11—N2'—C12'	0(100)
C3—C2—C7—C6	−54.6 (5)	C10—N2'—C12'—C13'	−62.7 (19)
Ni^iii^—O4—C8—O3	8.4 (8)	C11—N2'—C12'—C13'	112.6 (15)
Ni^iii^—O4—C8—C5	−169.0 (3)	N2'—C12'—C13'—C15'	−86 (2)
Ni^iv^—O3—C8—O4	−4.3 (5)	N2'—C12'—C13'—C14'	94.8 (19)
Ni^iv^—O3—C8—C5	173.0 (3)	C15'—C13'—C14'—C15'^v^	22 (4)
C4—C5—C8—O4	−154.6 (4)	C12'—C13'—C14'—C15'^v^	−159 (2)
C6—C5—C8—O4	−27.4 (5)	C14'—C13'—C15'—C14'^v^	−17 (3)
C4—C5—C8—O3	27.9 (5)	C12'—C13'—C15'—C14'^v^	163.9 (16)
C6—C5—C8—O3	155.1 (4)		

Symmetry codes: (i) −*x*+2, −*y*+1, −*z*+1; (ii) *x*+1, *y*, *z*; (iii) −*x*+1, −*y*+1, −*z*+1; (iv) *x*−1, *y*, *z*; (v) −*x*+1, −*y*, −*z*+2.

## References

[bb1] Batten, S. R. & Robson, R. (1998). *Angew. Chem. Int. Ed. Engl.* **37**, 1460–1494. 10.1002/(SICI)1521-3773(19980619)37:11<1460::AID-ANIE1460>3.0.CO;2-Z 29710936

[bb2] Brandenburg, K. (2006). *DIAMOND* Crystal Impact GbR, Bonn, Germany.

[bb8] Bruker (1997). *SMART* Bruker AXS Inc., Madison, Wisconsin, USA.

[bb9] Bruker (1999). *SAINT* Bruker AXS Inc., Madison, Wisconsin, USA.

[bb3] Kim, Y. J. & Jung, D.-Y. (2002). *Chem. Commun* pp. 908–909. 10.1039/b200658h 12123039

[bb4] Lee, S. W., Kim, H. J., Lee, Y. K., Park, K., Son, J.-H. & Kwon, Y.-U. (2003). *Inorg. Chim. Acta*, **353**, 151–158.

[bb5] Sheldrick, G. M. (1996). *SADABS* University of Göttingen, Germany.

[bb6] Sheldrick, G. M. (2008). *Acta Cryst.* A**64**, 112–122. 10.1107/S0108767307043930 18156677

[bb7] Yang, J., Ma, J.-F., Batten, S. R. & Su, Z.-M. (2008). *Chem. Commun.* pp. 2233–2235. 10.1039/b800199e 18463750

